# Tracing the adaptive evolution of SARS-CoV-2 during vaccine roll-out in Norway

**DOI:** 10.1093/ve/vead081

**Published:** 2023-12-20

**Authors:** Ignacio Garcia, Yunsung Lee, Ola Brynildsrud, Vegard Eldholm, Per Magnus, Anita Blomfeldt, Truls M Leegaard, Fredrik Müller, Susanne Dudman, Dominique A Caugant

**Affiliations:** Centre for Fertility and Health, Norwegian Institute of Public Health, 0213 Oslo, Norway; Division for Infection Control, Norwegian Institute of Public Health, 0213 Oslo, Norway; Division for Infection Control, Norwegian Institute of Public Health, 0213 Oslo, Norway; Centre for Fertility and Health, Norwegian Institute of Public Health, 0213 Oslo, Norway; Department of Microbiology and Infection Control, Akershus University Hospital, 1478 Lørenskog, Norway; Department of Microbiology and Infection Control, Akershus University Hospital, 1478 Lørenskog, Norway; Institute of Clinical Medicine, University of Oslo, 0316 Oslo, Norway; Institute of Clinical Medicine, University of Oslo, 0316 Oslo, Norway; Department of Microbiology, Oslo University Hospital, 0424 Oslo, Norway; Institute of Clinical Medicine, University of Oslo, 0316 Oslo, Norway; Department of Microbiology, Oslo University Hospital, 0424 Oslo, Norway; Division for Infection Control, Norwegian Institute of Public Health, 0213 Oslo, Norway; Department of Community Medicine and Global Health, Faculty of Medicine, University of Oslo, Blindern, 0316 Oslo, Norway

**Keywords:** COVID-19, phylogeny, mutations, vaccination

## Abstract

Vaccination against SARS-CoV-2 has greatly mitigated the impact of the COVID-19 pandemic. However, concerns have been raised about the degree to which vaccination might drive the emergence and selection of immune escape mutations that will hamper the efficacy of the vaccines. In this study, we investigate whether vaccination impacted the micro-scale adaptive evolution of SARS-CoV-2 in the Oslo region of Norway, during the first nine months of 2021, a period in which the population went from near-zero to almost 90 per cent vaccine coverage in the population over 50 years old. Weekly aggregated data stratified by age on vaccine uptake and number of SARS-CoV-2 cases in the area were obtained from the National Immunization Registry and the Norwegian Surveillance System for Communicable Diseases, respectively. A total of 6,438 virus sequences (7.5 per cent of the registered cases) along with metadata were available. We used a causal-driven approach to investigate the relationship between vaccination progress and changes in the frequency of 362 mutations present in at least ten samples, conditioned on the emergence of new lineages, time, and population vaccination coverage. After validating our approach, we identified 21 positive and 12 negative connections between vaccination progress and mutation prevalence, and most of them were outside the Spike protein. We observed a tendency for the mutations that we identified as positively connected with vaccination to decrease as the vaccinated population increased. After modelling the fitness of different competing mutations in a population, we found that our observations could be explained by a clonal interference phenomenon in which high fitness mutations would be outcompeted by the emergence or introduction of other high-fitness mutations.

## Introduction

Severe acute respiratory syndrome coronavirus 2 (SARS-CoV-2) has been responsible for the greatest pandemic of the past 100 years, with more than 600 million cases recorded worldwide and a global death toll that has passed 6.5 million people as of September 2022 (https://www.worldometers.info/coronavirus/). The spread of the virus has been tracked in near real-time using advanced genomic technology, mainly through sequencing of full-length viral genomes and the depositing of these sequences in a common database (https://www.gisaid.org). With over 10 million publicly available genomes at the time of writing, around 2 per cent of the official total number of COVID-19 cases, SARS-CoV-2 has quickly become the most intensely sequenced organism in history. This has allowed unparalleled insight into the virus evolution and has been significant to inform public health policies.

Soon after the onset of the pandemic, sets of fitness-enhancing mutations rapidly emerged and variants of concern with increased transmissibility were identified. With the implementation of COVID-19 vaccination programs toward the end of 2020, the virus was subjected to increased selection pressure. The vaccination occurred concomitantly with the propagation of the Delta variant lineage which rapidly replaced the Alpha and Beta variant lineages.

Norway had fairly low numbers of cases over the first two years of the pandemic, with roughly 50,000 cases in 2020 and 350,000 in 2021 in a population of 5.4 million (https://www.fhi.no/en/id/infectious-diseases/coronavirus/daily-reports/daily-reports-COVID19/). Whole-genome sequencing was an essential part of the molecular surveillance strategy from the start, with 44,419 high-quality full-length SARS-CoV-2 genomes from Norway published in GISAID in 2020 and 2021, which means that on average 1 in 9 diagnosed infections was fully sequenced and made publicly available. Vaccination, mainly with the mRNA vaccines, started on 27 December 2020. A strategy of vaccinating first the elderly and high-risk patient groups was adopted nationwide. A year later, the proportion of the population that had received at least two doses had plateaued at 73 per cent (https://www.fhi.no/en/id/vaccines/coronavirus-immunisation-programme/coronavirus-vaccination—statistic/). The impact of this vaccination strategy, mainly structured by age-groups, on the epidemiology of the pandemic in Norway has been demonstrated (Seppälä et al. 2020).

In the current study, we investigated amino acid changes in the virus during vaccine roll-out in the capital of Norway, Oslo, and the region surrounding it, Viken, where both virus sequences and patient data were available.

We were able to identify 362 mutations that were present in at least 10 of all sequenced viruses, of which 21 were positively associated and 12 were negatively associated with vaccination progression. Most of the positively associated mutations declined as vaccination progressed. By modelling the fitness of different viruses, we found that the most likely explanation for these observations was a clonal interference phenomenon driven by the subsequent introduction of new immune escape variants with higher transmissibility.

## Materials and methods

### Isolates and sequences

Virus samples collected in week 1 through 40 of 2021 (1 January to 5 October) from individuals living in Oslo, the capital city of Norway, or in municipalities in Viken, the region around Oslo, that had been sequenced either at Oslo University Hospital, Oslo (OUS), or Akershus University Hospital, Lørenskog (Ahus), were included in the study. Only virus genomes with a sequence length of 29,000 nt or longer published in GISAID (https://gisaid.org/hcov19-variants/) were used and a single unique sequence per infection was included. Anonymous patient data (age, gender, and municipality of residence) were provided by the two hospitals for these samples.

This resulted in a collection of 6,438 SARS-CoV-2 sequences, with 5,241 sequenced at OUS and 1,197 at Ahus. During that period, 39,668 cases were reported in Oslo (population 0.7 million) and 45,631 cases were reported in Viken (population 1.3 million) according to the Norwegian Surveillance System for Communicable Diseases (MSIS). Thus, our collection included 7.5 per cent of the recorded cases.

### Calculation of vaccination probability

Due to strict data protection policies, we could not retrieve vaccination status at the individual level. Therefore, to investigate how implementation of vaccination might impact the virus genome and select for mutants, we used data from the National Immunization Registry (SYSVAK) and from MSIS. Weekly aggregated numbers of SARS-CoV-2 cases stratified by municipality/city borough, age (in 5-year interval brackets), and gender were obtained from MSIS, while weekly aggregated data stratified over municipality/city borough, age, and gender on vaccine type and dose number were obtained from SYSVAK.

The SYSVAK data were used to calculate the weekly vaccination rate and the cumulative number of those who were fully vaccinated (two doses) by week for each of the following age-group: ≤6, 6–20, 21–35, 36–50, 51–65, 66–80, or ≥81 and for the two regions Oslo or Viken. Then, we divided the cumulative number by the total number of individuals for each age group and region, as obtained from Statistics Norway (https://www.ssb.no). The resultant numbers were referred to as ‘vaccination probability’ hereafter.

The statistical significance of the differences between the vaccination probability and the vaccination ratios in Oslo and Viken was calculated using Wilcoxon test in R (R Core Team, 2022). The *P*-values were adjusted using the Holm-Bonferroni method (Holm 1979).

### Virus sequence analyses

**Figure 1.**
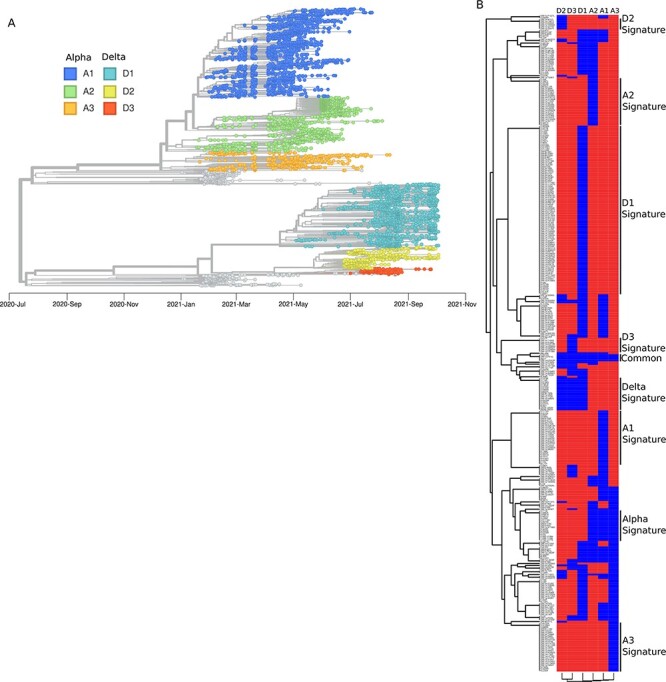
Phylogeny and mutation profiles of SARS-CoV-2 in Oslo and Viken (Norway) in 2021. (A) Phylogenetic tree of the SARS-CoV-2 isolated in Oslo and Viken between January and October 2021. The sequences were classified into several sub-lineages. The Alpha sequences were assigned to one of the three Alpha lineages: A1, A2, or A3. Similarly, the Delta viruses were assigned to the Delta lineages D1, D2, or D3 according to their phylogeny. (B) Mutation profile plot of Alpha and Delta lineages. The mutations present in ten or more sequences were extracted and assigned to the different lineages in which they were found. The mutations and lineages were ordered based on their similarity profiles. The signature mutations for the different lineages were also identified and labelled.

Samples were whole-genome sequenced using two multiplexed amplicon approaches: ARTIC-network nCoV-19 protocol v.3 (available at https://arctic.network/ncov-2019, accessed on 1 December 2021), run on either the Illumina MiSeq platform (Illumina Inc, San Diego, CA, USA) or the NanoPore GridIon (Oxford Nanopore Technologies, Oxford, UK); or the Swift Amplicon SARS-CoV-2 Panel (Swift Biosciences, Ann Arbor, MI, USA) on Illumina NovaSeq (Illumina Inc, San Diego, CA, USA) at the Norwegian Sequencing Centre, OUS, and at the Department of Microbiology and Infection Control, AHUS, according to the manufacturer’s instructions with minor modifications. We used the PANGO lineages nomenclature (https://github.com/cov-lineages/pangolin, accessed on 28 September 2021) to define the SARS-CoV-2 variant. Sequences were aligned in a codon-aware manner to the reference genome Wuhan-Hu-1 (GenBank accession no: MN908947.3) using the program Nextalign v.0.2.0 (available at https://github.com/nextstrain/nextclade, accessed on 1 December 2021), and a maximum-likelihood phylogenetic tree was created using IQTREE v.2.0.3 (available at https://iqtree.org, accessed on 1 December 2021). This phylogeny was used to define six subgroups in the virus population: Alpha 1, Alpha 2, Alpha 3, Delta 1, Delta 2, and Delta 3 (Fig. 1).

### Direct acyclic graph construction and causal modelling

To estimate the effects of the vaccination probability on the mutations, we constructed a direct acyclic graph (DAG) including the most likely elements and connections that could have a causal impact on the presence/absence of a mutation (Pearl 1995; Greenland, Pearl, and Robins 1999).

The nodes of the DAG represent the genotype of the virus and the factors that theoretically could affect the genotype. The paths represent the theoretical causal connections between the nodes, and the directionality of the paths represents the causality dependency between nodes (i.e. an arrow from A to B means that A is a cause of B). We estimated the strength of each of the paths by a linear mixed model approach using the lme4 (Bates et al. 2015) package in R . We generated 362 models, one per each of the mutations that were present more than 10 times in the dataset. For each model-mutation, the algorithm aimed to predict the presence/absence of the mutation, using the vaccine probability (fixed effect) and the week and age-group (random effect terms). Before running each model, we excluded the samples with sequencing drop-outs in the area where the mutation of interest was located. The coefficient estimates for the random effects and fixed effects were extracted. The significance of the random effects was adjusted using the Holm-Bonferroni method (Holm 1979). The 95 per cent confidence intervals (CI) for the coefficients of the fixed effects were calculated by bootstrapping the modelling (*N* = 500).

### Minimum spanning tree generation

To build the minimum spanning tree (MST), we first computed a distance matrix based on the DNA sequence using the ape package (Paradis, Schliep, and Schwartz 2019) in R. The MST was then generated using an optimized version of the *mst()* function from the *ape* package, *igraph* (Csardi and Nepusz 2006) and *ggnetwork* (Briatte 2021) packages (see the Github repository https://github.com/garcia-nacho/3VirusSim for the code).

### Calculation of prevalence of the different mutations and lineages

The prevalence of the different lineages and mutations for the different countries at different dates was obtained from the Outbreak.info database (which is based on GISAID sequences) using the R package *outbreakinfo* (Alkuzweny, Gangavarapu, and Hughes 2023).

### In silico competition assay

To understand which combination of parameters could lead to a scenario like the one that we observed in our analyses, we implemented an *in-silico* competition assay in which three viruses (early variant V1, late variant V2, and control variant V0) competed on a population consisting of vaccinated and unvaccinated individuals.

Each virus had three randomly assigned parameters to represent the ability to infect the vaccinated and unvaccinated and their transmissibility. For each simulation, the three viruses competed to saturation on nine scenarios where the vaccination rates ranged from 10 per cent to 90 per cent. The simulations were based on a four-compartment model: susceptible-vaccinated, susceptible-unvaccinated, infected-vaccinated and infected-unvaccinated, and were solved using ordinary differential equations. The transitions between susceptible and infected were modelled by the parameters assigned to the viruses and were encoded as coefficients of the equations that controlled the expansion of the viruses. We assumed fixed rates of vaccinated and no decay of the vaccine effect over time; therefore, no transitions between the vaccinated and the unvaccinated compartments were implemented. For each simulation, we computed the relative proportions of the viruses in the vaccinated and unvaccinated, together with the correlations between vaccination rates and the prevalence of the viruses.

The simulations in which V1 had a higher prevalence among the vaccinated and a negative correlation between its expansion and the vaccination rates were labelled as mimicking simulations and the parameters assigned to the three viruses were analysed. In total, we ran nine million simulations, consisting of one million combinations in nine vaccination scenarios. All the models were run in R using the *deSolve* package (Soetaert, Petzoldt, and Woodrow Setzer 2010) to solve the differential equations and the packages *doParallel* (Weston and Microsoft Corporation 2020a), *foreach* (Weston and Microsoft Corporation 2020b), *doSNOW* (Weston and Microsoft Corporation 2020c), and *parallel* to parallelize the simulations. See Supplemental Methods for the equations and parameters used in the simulations. The code to run the simulations is located in the Github repository https://github.com/garcia-nacho/3VirusSim.

To calculate the overall fitness of the competing viruses, we first calculated the overall fitness on the vaccinated by combining the basal fitness, the transmissibility on the vaccinated, and the immune escape fraction. Then, we multiplied the overall fitness on the vaccinated by ratio of vaccinated on the simulation and we added it to the basal fitness multiplied by the ratio of unvaccinated, since the overall fitness on the unvaccinated is equal to the basal fitness in our simulations.

### Statistical analyses

**Figure 2.**
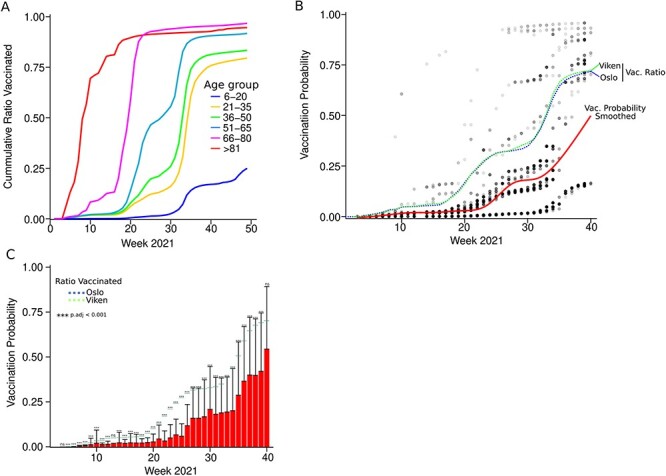
Comparison between SARS-CoV-2 vaccination ratio and vaccination probability. (A) Line plot showing the cumulative ratios of people vaccinated with at least one dose in Norway in 2021 according to their age. (B) Lines and scatter plot showing the calculated vaccination probability for each of the samples (dots). The smoothed cumulative vaccination probability was calculated (red line) and compared with the cumulative vaccination ratios in Oslo (blue dashed line) and Viken (green dashed line). (C) Bar plot showing the weekly vaccination probabilities (red bars) and the vaccination ratios in Oslo (blue dashed line) and Viken (green dashed line). Statistical significances between calculated probabilities and vaccination rates were calculated for Oslo and Viken using the Wilcoxon test and the *P*-values were adjusted using Holm-Bonferroni method. The error bars represent the standard deviation of the vaccination probabilities. *** represents adjusted *P*-value <0.001 and ns represents adjusted *P*-value >0.05.

All the statistical analyses were performed in R. All the plots were generated using the *ggplot2* package (Wickham 2016). Statistical significance for the results presented in Figs 2C, 4B, 4C, and 4D was calculated using Wilcoxon test due to the non-parametric nature of the data. The significance of the correlation between prevalence and full vaccinated ratios for the European countries (Fig. 4E and F) was calculated using the *F-statistic* after fitting a linear model with the *lm()* function in R.

## Results

### Phylogenetic analysis

We collected 6,438 whole-genome sequences from virus samples isolated in Oslo and Viken during the first 40 weeks of 2021, which is when most of the population was vaccinated in Norway. A phylogenetic tree of the sequences was constructed using Nextstrain (Hadfield et al. 2018) clearly showing the successive occurrence of the Alpha and Delta lineages (Fig. 1A). Upon visual inspection of the phylogenetic tree, we identified three internal clusters within each of the Alpha and Delta main lineages that we named A1, A2, A3, D1, D2, and D3 (Fig. 1A). A total of 5,757 virus sequences (or 89.4 per cent of the samples) belonged to the 6 sub-lineages. A1, A2, A3, D1, D2, and D3 contained 2,019, 1,232, 437, 1,436, 494, and 139 sequences, respectively. We could not detect strong differences on the distribution of these six sub-lineages between Oslo and Viken (Fig. S1A). We focused our analysis on these six clusters by filtering out the non-Alpha and non-Delta sequences.

### Mutation analysis

The mutations present in the 5,757 sequences were extracted using Netxclade (Aksamentov et al. 2021). To avoid the collection of spurious mutations due to sequencing errors, we selected the mutations (substitutions and deletions) that were present in at least 10 sequences, and we identified 362 such mutations. The distribution of these mutations in each of the six sub-lineages is shown in Fig. 1B. Only four mutations were present in all six sub-lineages: 28271, ORF1a:K3353R, ORF1b:P314L, and S:D614G.

The S:D614G substitution in the Spike protein that has been shown to provide increased transmission and infectivity (Long et al. 2020) and that arose in early 2020 and became dominant worldwide (Korber et al. 2020) was present in all samples in our dataset. ORF1b:P314L, a mutation that has been shown to co-occur with S:D614G (Ogawa et al. 2020), was present in 99.97 per cent of the samples and almost all the samples (99.91 per cent) had one nucleotide deletion at position 28271. This deletion overlaps with the promoter of the N gene and it has been proposed to alter the expression levels of the genes N and ORF9 by affecting the Kozak site of N (Yang et al. 2021; Thorne et al. 2022). Finally, the ORF1a:K3353R mutation was present in all the six sub-lineages, but only 2.1 per cent of the sequences harboured it, suggesting that although it is easily acquired, it does not confer a significant fitness advantage to the virus.

We identified 17 mutations that were specific of the Alpha sequences, i.e. they were found in all three Alpha sub-lineages and did not occur among Delta sequences (Fig. 1B). These Alpha-signature mutations (11288–11296, 21765–21770, 21992–21994, S:N501Y, S:P681H, S:S982A, S:T716I, S:L938F, S:A570D, S:D1118H, ORF8:R52I, ORF8:Y73C, ORF1a:A1708D, N:D3L, N:G204R, N:R203K, N:S235F), with the exception of S:L938F, were present in more than 99.3 per cent of the Alpha sequences. Of the 18 Delta-signature mutations that we identified, 16 were present in at least 96.0 per cent of all the Delta sequences (22029–22034, 28248–28253, M:I82T, N:D377Y, N:D63G, N:R203M, ORF1b:G662S, ORF7a:T120I, ORF7a:V82A, ORF9b:T60A, S:A222V, S:D950N, S:G142D, S:L452R, S:P681R, S:R158G, S:T19R, S:T478K). The two exceptions were S:A222V and S:G142D, which accounted just for 29.7 per cent and 72.5 per cent of Delta sequences, respectively.

We also found mutations that were sub-lineage specific, i.e. they occurred only in one sub-lineage (Fig. 1B). Only one of the A1 specific mutations (ORF1b:P1001S) was present in more than 90 per cent of the A1 sequences and there were no specific mutations for the sub-lineages A2 and A3. Sub-lineage D1 had eight specific mutations (N:G215C, ORF1a:A1306S, ORF1a:P2046L, ORF1a:P2287S, ORF1a:T3255I, ORF1a:T3646A, ORF1a:V2930L, ORF1b:A1918V), D2 had three (ORF1b:D260G, ORF1b:I1887V, ORF1a:I2541V), and D3 had none.

### Computing SARS-CoV-2 vaccination probability

Vaccination in Norway was stratified by age-groups, with the older and people at risk being vaccinated first (Fig. 2A). Using the information about the weekly vaccination count for each age group in Oslo and Viken registered by SYSVAK, we computed the vaccination probability for each of the patients from whom samples were sequenced (Fig. 2B) (see Supplemental Methods for details). We noticed that the average vaccination probability assigned to the samples on our dataset was significantly lower than the overall nationwide vaccination ratio, suggesting that there was an enrichment of samples from unvaccinated individuals in our dataset (Fig. 2C). This finding supports the fact that vaccination reduced the incidence of SARS-CoV-2 during the Alpha and Delta waves, in agreement with studies about the efficacy of SARS-CoV-2 vaccination (Ghazy et al. 2022; Graña et al. 2022).

### Inference models can be used for fine-grained epidemiological analyses

**Figure 3.**
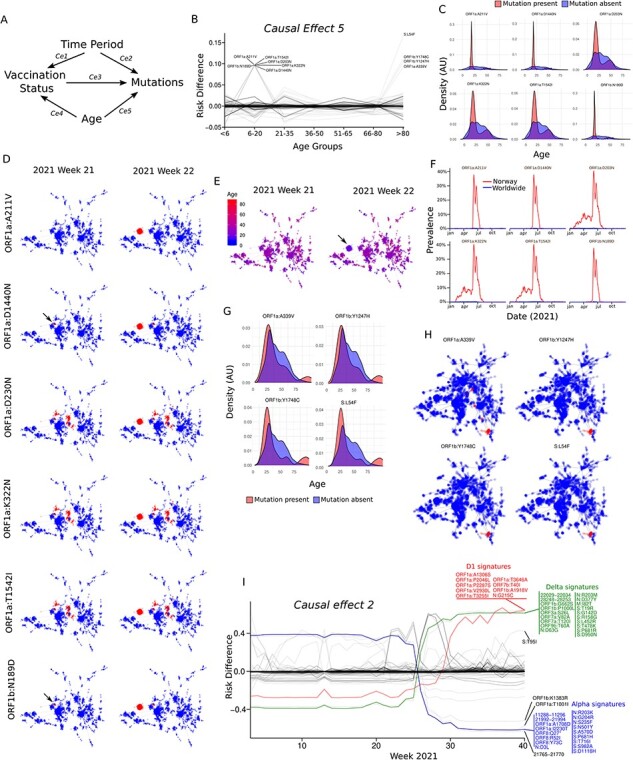
Causal relationships between date, age, and SARS-CoV-2 mutations. (A) DAG representing five causal effects (Ce1 to Ce5) (see main text for details). (B) Line plot showing the causal effect 5. The risk difference for each mutation within each age group was calculated and plotted as a line. The mutations with a higher risk difference than 0.05 were labelled. (C) Density plots showing the age of the patients carrying the mutations with a Ce5 risk difference larger than 0.05 for the age-group 6–20. (D) Cumulative minimum spanning trees showing the relationships and relative distances between the sequences present from weeks 1–21 (labelled as 2021 week 21) or 1–22 in 2021 (labelled as 2021 week 21). The sequences containing each of the mutations with a Ce5 risk difference larger than 0.05 for the age group of 6–20 years were labelled as red points. The arrow shows the sequences containing the mutation ORF1a:D1440N on week 21. (E) Cumulative spanning tree showing the age of the sequences associated with the *russ*-outbreak (arrow). (F) Line plots showing the relative incidence of the six mutations with a Ce5 risk difference larger than 0.05 for the age group of 6–20 years both in Norway (red line) and worldwide (blue line). (G) Density plots showing the age of the patients carrying the mutations with a Ce5 risk difference larger than 0.05 for the age-group >80. (H) Minimum spanning tree of the entire dataset; the mutations with Ce5 risk differences larger than 0.05 for the age-group >80 were labelled as red dots. (I) Line plot showing the causal effect 2. The risk difference for each mutation during each week was calculated and plotted as a line. The mutations with Ce2 risk differences larger than 0.4 at week 40 were labelled. The mutations with overlapping profiles were assigned to each of the one of the following signature groups: Alpha, Delta, or D1.

The main hypothesis of our study was that a causal relationship existed between the SARS-CoV-2 vaccination program in Norway and the emergence of some mutations. However, the use of uncorrected statistical correlations might lead to false conclusions through the identification of spurious connections. As most of the population was vaccinated in Norway at a time when most of the circulating viruses were of the Delta lineage (Fig. 2A, S1B and S1C), a strong statistical correlation was expected between Delta-specific mutations and their presence in samples from vaccinated individuals, although there is no evidence supporting a causal relationship between the increase of the Delta variant and the vaccination programs. To tackle this problem, we used a causal inference approach. First, we constructed a direct acyclic graph (DAG) (Pearl 1995; Greenland, Pearl, and Robins 1999) to dissect the causal relationships between vaccination status, time-period, age of the patient, and the presence of certain mutations (Fig. 3A).

We accounted for five possible causal effects (*Ce1* to *Ce5*). *Ce1* is the effect of time on the vaccination status (i.e. the vaccination probability that we previously calculated) which is already known for Norway. By the time when the last sample of our dataset was taken, on 4 October 2021, 77.5 per cent of the population had received one dose and 68.1 per cent two doses of a COVID-19 vaccine (Mathieu et al. 2020) (Fig. S1B). *Ce2* is the effect of time on the presence of different mutations. *Ce2* aggregates several effects, such as virus import ratios (Osnes et al. 2021), border control policies, mutation rates, natural selection, and probably others. Although *Ce2* is different from zero, its range and sign is unknown. *Ce3* is the effect of the vaccination status as a driving force for changes in the frequencies of certain mutations. The main hypothesis of this study is that *Ce3* is significantly different from zero for several mutations. *Ce4* is the effect of age on the vaccination status which is also known for the Norwegian population because the vaccination program was stratified into age-groups that were vaccinated consecutively (Fig. 2A) (Mathieu et al. 2020). Finally, *Ce5* is the effect of age on the presence of certain mutations. There are two possible effects that can be bundled into *Ce5*: (1) Differences in the affinities of certain mutations for the different age-groups and/or (2) the presence of age-specific outbreaks (e.g. outbreaks in daycare centres, schools, nursing homes, etc).

By looking at the DAG it is possible to identify two true confounders: age and time-period, since they control mutations directly and through the vaccination status. Therefore, to block any *causal back-door* (Pearl 2009; Brumback 2021), a linear mixed model approach was used with the vaccination probability as the sole covariate and age and week of infection as random effects.

After fitting the 362 generated models, we verified whether they agreed with the epidemiological data at the time. We first studied the random effects associated with the different age-groups (i.e. *Ce5*) and observed that the coefficients for ten mutations for two age-groups (6–20 and >80 years) had a risk difference larger than 0.05 (Fig. 3B). Especially, the sequences containing the mutations linked to the age-group 6–20 years (ORF1a:A211V, ORF1a:D1440N, ORF1a:D203N, ORF1a:K322N, ORF1a:T1542I, ORF1b:N189D) showed a peak in patients around 18 years old (Fig. 3C). Five of these six mutations were restricted to the A2 sub-lineage, while ORF1a:D203N was present in both A2 and D1 sub-lineages.

To study these mutations over time, we constructed a minimum spanning tree (MST) and dissected it weekly (see Methods for details). By doing this, we could investigate whether the age difference for the six 6-to-20-years-mutations was the result of an age-specific outbreak or the preferential affinity of the mutations for this age-group. We found that these six mutations were strongly associated with a large outbreak starting in week 22 (Fig. 3D). The average age of the patients in the outbreak was significantly lower (*P*-value = 1.7 10^–11^) than the rest of the patients whose viruses were collected during the same week (Fig. 3E). In total, 151 virus sequences were assigned to the outbreak and the average age of the patients was 20.04 years (SD = 7.78), while the mean age of the 131 patients outside the outbreak during the same week was 30.18 (SD = 14.9). This large outbreak was due to a celebration of the students graduating from secondary school (‘*russ*’ celebration https://en.wikipedia.org/wiki/Russefeiring), a controversial event that was highly covered by the Norwegian mass media at the time. The six mutations connected to the *russ-outbreak* had distinct patterns on the MST. While ORF1a:D203N, ORF1a:K322N, and ORF1a:T1542I were already widely distributed in Norway (212 sequences in Oslo and the surrounding region by week 21), ORF1b:N189D and ORF1a:D1440N were connected with a small outbreak of 8 sequences in week 21 (Fig. 3D). In contrast, the ORF1a:A211V mutation only occurred in samples connected with the outbreak in week 22. All these six mutations were quite specific to Norway; the maximum prevalence in Norway was 40.4 per cent while the maximum prevalence worldwide was 0.5 per cent (Fig. 3F).

Similarly, the four mutations with higher *Ce5* risk-difference on the >80 age-group (ORF1a:A339V, ORF1b:Y1247H, ORF1b:Y1248C, and S:L54F) were present on sequences from patients older than 80 years (Fig. 3G). The distribution of these mutations on a global MST revealed that they were connected to an outbreak (Fig. 3H), probably happening in a nursing home. Altogether, these results show that the models can identify correctly the mutations linked with age-specific outbreaks and to take this into account when computing the causality connections.

### Effect of time on the distribution of SARS-CoV-2 mutations

*Ce2* (i.e. the effect of time on the presence of different mutations) was investigated by analysing the random effects associated with the different weeks. Three groups of mutations with similar overlapping profiles were identified (Fig. 3I, red, blue, and green lines). When connecting these mutations with those previously identified as signatures of the different lineages/sub-lineages, we found that the group of mutations that increased the risk-difference after week 25 (Fig. 3I, green line) was present in all three Delta sub-lineages (D1, D2, D3) (Fig. 1B). Indeed, all the Delta signatures on the Spike protein (S:T19R, S:L452R, S:T478K, S:P681R, and S:D950N) were included in this group. We identified two additional spike mutations on the all-Delta cluster (S:G142D and S:R158G).

Looking at the mutations associated with decreasing risk-difference after week 25 (Fig. 3I, blue line), we found that all of them were present in the Alpha sub-lineages A1, A2, and A3, and that all the Alpha-signature mutations on Spike were included (i.e. S:N501Y, S:A570D, S:P681H, S:T716I, S:S982A, and S:D1118H). Thus, our model was able to identify the replacement of Alpha lineages by Delta lineages starting on week 25 in 2021 and *Ce2* correctly accounted for it.

Finally, we investigated the group of mutations with an increased risk-difference after week 30 (Fig. 3G, red line). Eight (N:G215C, ORF1a:A1306S, ORF1a:P2046L, ORF1a:P2287S, ORF1a:T3255I, ORF1a:T3646A, ORF1a:V2930L, ORF1b:A1918V) of the nine mutations found in that group were indeed the D1-signature mutations. This suggested that the D1 lineage was introduced and expanded in Norway when other Delta lineages were already present in the country.

Altogether, these results showed that our causal models are strongly supported by the epidemiological data, thus validating the use of causal inference approaches for studying the effect of vaccination on the mutations.

### The mutations more prevalent in the vaccinated declined as the vaccination increased

**Figure 4.**
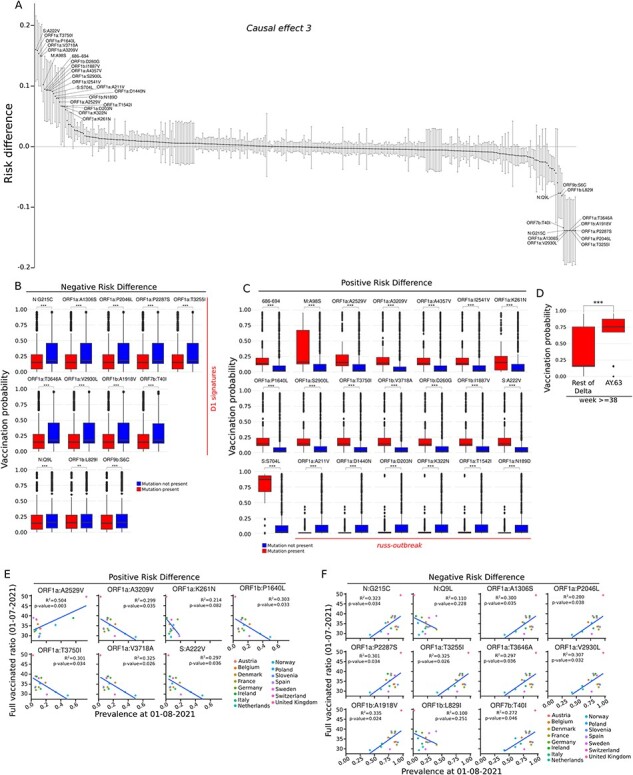
Causal relationships between vaccination probability and SAR-CoV-2 mutations. (A) Waterfall plot representing the strength of the causal effect 3 (i.e. the causal relationship between vaccination and SARS-CoV-2 mutations) for each mutation. The mutations are represented as points and the error bars represent the 95 CI of the risk difference. The mutations with risk differences significantly larger than zero were labelled. (B) Boxplots showing the vaccination probability assigned to the samples carrying the mutations with a Ce3 risk difference significantly different from zero and lower −0.05. The statistical significances were calculated using the Wilcoxon test. The D1-signature mutations were highlighted. (C) Boxplots showing the vaccination probability assigned to the samples carrying the mutations with a Ce3 risk difference significantly different from zero and higher than 0.05. The statistical significances were calculated using the Wilcoxon test. (D) Boxplots showing the vaccination probability of the samples classified as AY.63 according to the Pangolin nomenclature, against the rest of delta viruses. The statistical significance was calculated using the Wilcoxon test. (E) Scatter plots showing the correlation between the incidence of the mutations with significant positive Ce3 risk difference and the vaccination ratios of different European countries. The lines represent the best fitting linear model for each mutation. The R^2^ of the fit and the *P*-value were shown. (F) Scatter plots were calculated as described in Fig. 4E but for the mutations with significant negative Ce3 risk difference. *** represents *P*-value <0.001, ** represents 0.001 < *P*-value < 0.01, * represents 0.01 < *P*-value < 0.05, ns *P*-value >0.05.

Then, we analysed the mutations that were causally connected with the vaccination progression (i.e. *Ce2*). A total of 21 mutations with risk-differences significantly different from zero and larger than 0.05 (Table 1) and 12 mutations with risk-differences significantly lower than −0.05 were identified (Table 2) (Fig. 4A). All the mutations with negative risk-differences were D1-specific mutations, suggesting that the set of Delta mutations that expanded in Norway from week 30 were predicted to have a negative causal relationship with vaccination. The vaccination probabilities of these samples with D1-specific mutations were indeed significantly lower (Fig. 4B), validating the approach. Among the 21 mutations with positive risk differences, six were connected with the *russ-outbreak* (Fig. 4C) and had a lower vaccination probability, as the outbreak occurred in an age-group that was not vaccinated at the time. The other 15 mutations with positive risk-differences were found in samples from individuals with higher vaccination probabilities (Fig. 4C) and were heterogeneously distributed within the Alpha and Delta sub-lineages (Table 1, Fig. 1B). Most samples carrying these mutations were assigned to the Delta Pangolin lineage AY.63 (Rambaut et al. 2020) (Fig. S1D). Comparing the vaccination probabilities between the AY.63 samples and the rest of the Delta lineage, we found that the average vaccination probability was significantly different after week 38 (Fig. 4D). Eight of these 15 mutations were identified in at least one Alpha sequence in our dataset. Moreover, although S:A222V was restricted to Delta lineages in our dataset, it has been frequently found in B.1.1.7 viruses worldwide (Fig. S1E). This lineage heterogeneity suggests that the nine mutations, 686-694, ORF1a:A2529V, ORF1a:A3209V, ORF1a:A4357V, ORF1a:P1640L, ORF1a:S2900L, ORF1a:T3750I, S:A222V, and S:S704L, provide increased viral fitness and therefore have a tendency to create homoplasies. Surprisingly, only two of these mutations were found on the Spike gene. Although neither S:A222V nor S:S704L show any antibody escape property (Ginex et al. 2022; Wang et al. 2022), both have been predicted to be high-fitness mutations (Wang et al. 2023). Indeed, S:S740L, the mutation with greater differences in the vaccination probability, arose again in 2022 connected with several Omicron sub-lineages and it is estimated that S:S704L increased the growth advantage of the baseline BA.2 lineage by a 35 per cent (Althaus et al. 2023).

**Table 1.** Mutations with positive Ce5 risk differences.

Mutation	Count	Risk difference	Lineages
686-694	512	0,094781	A1/A2/D1/D2/D3
M:A98S	120	0,102224	D1/D2
ORF1a:A211V	444	0,083235	A2
ORF1a:A2529V	240	0,073672	A1/A3/D1/D2
ORF1a:A3209V	625	0,149602	A1/D1/D2/D3
ORF1a:A4357V	501	0,092928	A1/A2/D1/D2
ORF1a:D1440N	453	0,079869	A2
ORF1a:D203N	688	0,066597	A2/D1
ORF1a:I2541V	455	0,090983	D2
ORF1a:K261N	186	0,061001	D1
ORF1a:K322N	687	0,066680	A2
ORF1a:P1640L	641	0,153417	A2/D1/D2/D3
ORF1a:S2900L	504	0,092882	A1/D1/D2
ORF1a:T1542I	679	0,067040	A2
ORF1a:T3750I	609	0,158775	A2/D2/D3
ORF1a:V3718A	623	0,149615	D2/D3
ORF1b:D260G	494	0,093367	D2
ORF1b:I1887V	494	0,093367	D2
ORF1b:N189D	453	0,079816	A2
S:A222V	614	0,160143	D1/D2/D3
S:S704L	30	0,087399	A2/D2

**Table 2.** Mutations with negative Ce5 risk differences.

Mutation	Count	Risk difference	Lineages
N:G215C	1,435	−0.138357	D1
N:Q9L	337	−0.076867	D1
ORF1a:A1306S	1,436	−0.138440	D1
ORF1a:P2046L	1,436	−0.138484	D1
ORF1a:P2287S	1,435	−0.138638	D1
ORF1a:T3255I	1,436	−0.138484	D1
ORF1a:T3646A	1,436	−0.138483	D1
ORF1a:V2930L	1,436	−0.138472	D1
ORF1b:A1918V	1,436	−0.138484	D1
ORF1b:L829I	300	−0.081200	D1
ORF7b:T40I	1,438	−0.133962	A3/D1/D2
ORF9b:S6C	337	−0.076867	D1

If the expansion of the mutations that we identified was causally connected to the SARS-CoV-2 vaccination program in Norway, we would expect that their prevalence should be somehow correlated with the vaccination progress in other countries as well. To test this hypothesis, we selected eighteen mutations with positive and negative risk-differences that were present in Norway and other European countries and computed the correlation between the vaccination rate and their prevalence (Fig. 4E and F). We used the percentage of fully vaccinated population by 1 July 2021 as a proxy for vaccination rate and the prevalence of the different mutations by 1 August 2021 in the 15 European countries with most sequences deposited in the international SARS-CoV-2 database GISAID (Khare et al. 2021). We found that the prevalence of six of the seven mutations with positive risk differences showed negative correlations with the vaccination ratios. Only one mutation (ORF1:A2529V) displayed a significant positive correlation between prevalence and vaccination status (Fig. 4E). For nine of the 11 mutations with negative risk difference, a significant positive correlation between the vaccination progression and the expansion of the mutation was seen (Fig. 4F), in agreement with our observations for Norway.

### In-silico competition assays suggest that the mutations causally connected with vaccination were replaced by variants with higher fitness

**Figure 5.**
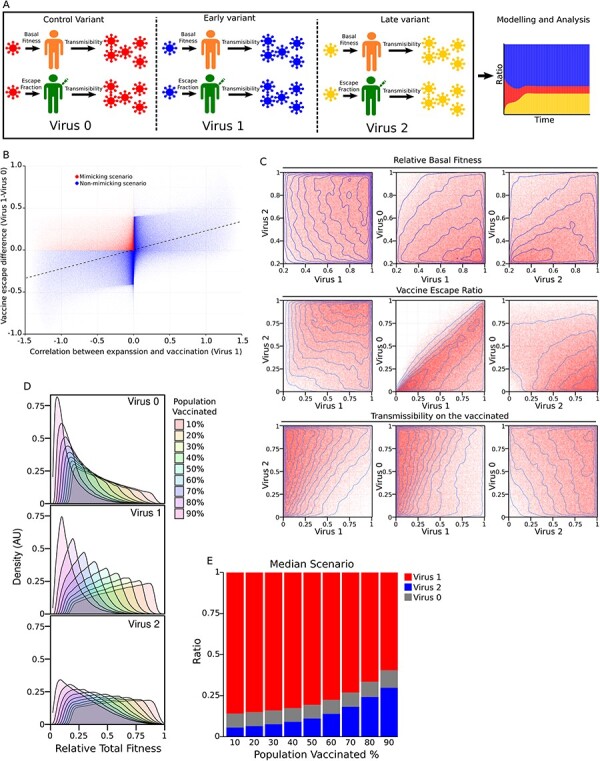
*In-silico* competition assay. (A) Visual representation of the competition assay. The competition involved three variants (A control Variant V0, an early variant V1 introduced together with V0, and a later variant V2, introduced later than V0 and V1). The fitness of each variant was defined by three independent parameters (basal infectivity, vaccine escape fraction, and transmissibility). (B) Scatter plot showing the relationship between the vaccine escape capability of V1 (i.e. the difference in the ability of V1 to infect the vaccinated when compared with V0) versus the slope of the line that correlate the expansion of V1 and the vaccination ratio (i.e. the *x-axis* of the scatter plot is the *SlopeV1* term of the equation ‘*RatioV1 = SlopeV1· VaccinationRate + IntersectV1ʹ*). The dashed line represents the linear regression model that better fits all the simulations. The plot shows the results for 1 million simulations where the simulations that mimic our observations *‘in vivo’* (i.e. scenarios in which larger vaccine escape capabilities of V1 correlate with a decrease in the expansion of V1 as the vaccinated fraction increases) are the red dots. (C) Scatter and contour plots for all the comparisons of the basal fitness, vaccine escape, and transmissibility for V0, V1, and V2. (D) Density plots showing the total fitness of V0, V1, and V2 for nine different vaccination scenarios in which a percentage of the population ranging from 10 per cent to 90 per cent had been vaccinated. (E) Bar plots showing the ratios of the different variants for different vaccination ratios (10 per cent–90 per cent) when the simulation used the median basal fitness, vaccine escape, and transmissibility coefficients for V0, V1, and V2 found on the mimicking scenarios.

To explore under which scenarios the expansion of the mutations more prevalent in the vaccinated could decrease as the vaccination rate increased, we devised an *in-silico* model in which we simulated the expansion and competition of three variants: a variant control (V0), an early variant (V1) introduced at the same time as the control variant, and a late variant (V2) introduced later than V0 and V1 (Fig. 5A).

For each simulated virus, the total fitness was decomposed into three coefficients: the basal fitness of the virus (i.e. its ability to infect the vaccinated), the vaccine escape fraction (i.e. how much of the basal fitness of the virus is retained on an encounter with a vaccinated individual), and the transmissibility (i.e. the probability of a virus to infect other individuals after the infection) (secondary attack rate) (Fig. 5A) for vaccinated and unvaccinated. We considered the expansion of the viruses for different ratios of vaccination ranging from 10 per cent to 90 per cent of the population vaccinated. We used a modified SIR (Susceptible-Infected-Recovered) model in which the susceptible group was divided into vaccinated and unvaccinated with predefined vaccination rates (see Methods and Supplementary methods for details).

We run one million *in-silico* competitions in which the aforementioned coefficients were randomly assigned for the three viruses. Then, we studied which simulations mimic our observations for Norway and elsewhere in Europe (i.e. those simulations in which V1 has a higher vaccine escape capability than V0 but the correlation between the prevalence V1 and the ratio of vaccinated decreases). To do that, we first computed the slope of the correlation between the expansion of the virus V1 and the ratio of vaccination in the population. A positive slope means that the variant expands as more people in the population are vaccinated. A negative slope means that the ratio of the variant shrinks in favour of other variants as more people get the vaccine. When we plot this V1-vaccine correlation (Fig. 5B, *x-axis*) versus the vaccine escape difference of V1 and V0 (Fig. 5B, *y-axis*), we found that, in general, as the vaccine escape fraction increased for the early variant V1, there is a positive V1-vaccine correlation (Fig. 5B, dashed line). However, a very significant fraction of the simulations shows the same phenomena that we have observed in our data (Fig. 5B, red dots).

Next, we studied the combinations of the coefficients of these mimicking simulations and we found that on scenarios:

1. The basal fitness of V1 and V2 tend to be higher than the basal fitness of the control variant. In 68.1 per cent of the mimicking scenarios, the basal fitness of V1 is larger than the one of V0, and in 65.6 per cent of the mimicking scenarios, the basal fitness of V2 is larger than V0. However, the difference between the basal fitness of V1 and V2 does not seem to be critical for the mimicking scenarios to happen. Only 55 per cent of the mimicking scenarios happen with the basal fitness of V1 being larger than the one from V2 (Fig. 5C upper panel). As expected, when comparing any two coefficients (e.g. basal fitness V1 vs basal fitness V0) from a large random distribution of values, approximately 50 per cent of the times one of them will be larger than the other one and therefore differences from 50 per cent can be interpreted as tendencies in the relationships between the coefficients to result in a mimicking scenario.
2. The vaccine escape capabilities of V1 and V2 are higher than the vaccine escape of the control variant. 87.5 per cent and 73.5 per cent of the mimicking scenarios showed a higher vaccine escape of V1 and V3 over V0, respectively, while only 55 per cent of the scenarios have larger vaccine escape fraction of V3 over V2 (Fig. 5C, medium panel).
3. The transmissibility on the vaccinated of the V1 is lower than the transmissibility on the vaccinated of V2 and even the transmissibility on the vaccinated of the control virus V0.

In 79 per cent and 75 per cent of the mimicking scenarios the transmissibility of V2 and V0 are larger than the one of V1, respectively. However, only in 54 per cent of the mimicking the transmissibility of V2 is higher than the transmissibility of V0 (Fig. 5C, lower panel).

Then, we calculated the overall fitness of the different strains under different vaccination ratios by combining basal fitness, vaccine escape, and transmissibility of the mimicking scenarios (see Methods) (Fig. 5D). Although we found that, as expected, the overall fitness of all the variants decreased as the vaccination ratio increased, the overall fitness of V2 is larger than the one of V1 and V0 under all vaccination rates. We computed the expansion of the three variants using the median of the basal fitness, vaccine escape, and transmissibility in the vaccinated coefficients of the mimicking scenarios. We observed that the median combination of coefficients produced a scenario in which the decline of V1 is caused by the expansion of the later variant V2. Finally, we evaluated how many of the mimicking scenarios were the result of the expansion of V2 over V1. We found that 95.4 per cent of the mimicking scenarios were the consequence of the later variant V2 taking outcompeting V1.

Overall, these results suggest that the most likely explanation for our observations is the raise of a later variant (V2) with higher fitness outcompeting other vaccine escape variants (V1).

## Discussion

Vaccination can drive the evolution of a virus by exerting selection pressure on the viral population. The first generation of SARS-CoV-2 vaccines works by eliciting an immune response that targets the Spike protein. This can create selective pressure on the virus to evolve to escape recognition by the immune system. The emergence of immune-escape mutations on the Spike protein in Omicron lineages is an evidence of this phenomenon (Willett et al. 2022). However, other mechanisms can help the virus to escape the immune system of the vaccinated (Chakarborty et al. 2022). Unfortunately, these other mechanisms, such as interferon dysregulation, natural killer cytotoxicity modulation, or increased viral assembly, cannot be easily evaluated with methods like antibody-binding affinities and, therefore, much less is known about them.

In this paper, a causal-driven approach was used to investigate the relationship between vaccination and changes in the frequency of different mutations. By studying the correlations between mutations and age-groups or date of infection, we were able to validate our approach and identify age-related outbreaks, such as the *russ-outbreak* that occurred in Norway in the spring of 2021, and an outbreak within the >80 age group in the fall of 2021.

Our models capture the replacement of Alpha lineage by Delta in 2021 and we are able to detect the spread of a Delta sub-lineage that we called D1 that became dominant in Norway after week 30. Furthermore, we identified twenty-one positive and twelve negative causal connections between vaccination and mutation prevalence (Tables 1 and 2). All the mutations with negative risk-difference were found in lineages that expanded as vaccination increased and almost all the mutations with positive risk-difference decreased as the vaccination program progressed. Interestingly, most of the mutations that we identified occurred outside the Spike coding gene and those that occurred in the Spike did not show antibody escape properties. This suggests that the vaccination roll-out did not favour the introduction of mutations with antibody escape properties.

A possible explanation for our observations is clonal interference. Clonal interference takes place when several beneficial mutations occur at different positions of the viral genome simultaneously in the population. These mutations interfere with each other as they compete for the human hosts (Strelkowa and Lässig, 2012). Models of SARS-CoV-2 showed that, during the pandemic, the ratio at which these high fitness mutations can occur is consistent with the clonal interference phenomena (Rouzine and Rozhnova 2023).

The results from our *in-silico* competition simulations support the clonal interference hypothesis. We found that the most likely scenario is the one in which late mutations with very-high transmissibility and immune escape capabilities took over previous high-fitness variants. These new mutations would have a much higher relative fitness at higher vaccination rates and therefore would expand quicker that the other variants as vaccination increases, leading to the observed phenomena. However, other scenarios in which the vaccine-escape mutations have a high cost in transmissibility on the vaccinated, although less likely, are also possible.

The fact that the mutations that we identified with positive risk-difference tend to form homoplasies and that have occurred later on in the pandemic also supports the idea that they provide a fitness increase to the virus. It is logical to speculate that the very-high fitness mutations that took over them were mutations with antibody escape capabilities. Unfortunately, our causal models could not identify them because of the time limitation of our dataset.

While the causal-driven approach used in this study has provided valuable insights into the relationship between vaccination and changes in the frequency of different mutations, it is important to acknowledge the challenges associated with using causal inference in this type of research. One of the main challenges is the potential for confounding variables to influence the results. We attempted to control for confounding factors by adjusting for age and date; however, there may be other unmeasured confounders that could have influenced the results, such as changes in public health measures or variations in the prevalence of different variants across different regions. Another possible confounder is the import of variants during the summer vacations of 2021 which is highly aligned with large changes on the vaccination ratios.

Another challenge is the potential for selection bias, as individuals who choose to get vaccinated may differ systematically from those who do not, making it difficult to infer causality. Moreover, our approach to compute the vaccination probability is not complete since it did not account for the prioritization of the vaccination of health workers and other high-risk groups. Without data on vaccination at the individual level, it would be impossible to account for those confounders.

Finally, we would like to acknowledge the possibility that the linear-mix model approach that we used was too simple and that some of the confounders/factors had non-linear connections between them that were not explicitly modelled by our approach. However, including extra non-linear terms in the analysis would reduce the interpretability of the coefficients of the model and therefore it would be very difficult to estimate any causal effect from the model.

Due to complex epidemiological confounding, it remains unclear whether the mutations identified in this study affect transmission in a vaccine status specific manner and additional studies including more data together with *in-vitro* analyses would be required to draw conclusions about the role of the mutations that we identified on the biology of the virus.

Despite these challenges, the causal-driven approach used in this study represents an important step forward in our understanding the evolution of SARS-CoV-2. By acknowledging these limitations and continuing to refine this method, scientists can continue to build upon these findings to inform effective public health strategies to combat future pandemics.

## Supplementary Material

vead081_Supp

## Data Availability

All the viral sequences can be accessed using their GISAID ID present on the Supplementary Table 2. The metadata will be provided upon request.
