# Supplementary material for: Tracing the adaptive evolution of SARS-CoV-2 during vaccine roll-out in Norway
Source: Virus Evol. 2023 Dec 20;10(1):vead081. doi: 10.1093/ve/vead081 (PMC10776306; doi:10.1093/ve/vead081)
Supplement: vead081_Supp [file vead081_supp.zip › suppl_data/SupplementalMethods_text.docx]

**Supplemental Methods.**

Equations for the vaccine probability calculation:

Given the following age groups, regions, weeks, populations per age group and vaccination counts:

$$AgeGroup=\left\{ {AG}_{1}, ..,{AG}_{n} \right\}$$

$$Region=\left\{ R_{1}, ..,R_{r} \right\}$$

$$Week=\left\{ W_{1}, ..,W_{p} \right\}$$

$$Population=\left\{ P_{R_{1};{AG}_{1}}, ..,P_{R_{r};{AG}_{n}} \right\}$$

$$VaccinationCount=\left\{ {VC}_{{AG}_{1};R_{1};W_{1},}, ..,{VC}_{{AG}_{n};R_{r};W_{p},} \right\}$$

The vaccination probabilities were computed for each age-group, region and group:

$${VaccineProbability}_{{AG}_{i};R_{j};W_{k}}=\frac{\sum_{week=1}^{k} {VC}_{{AG}_{i};R_{j};W_{week}}}{P_{R_{j};{AG}_{i}}}$$

These vaccination probabilities were assigned to each of the samples by using the metadata of the sample (I.e., age-group, region, week)

Given that:

${Sample}_{s}:(AgeGroup=i;Region=j;Week=k$)

Then:

$${{VaccinationProbability}_{Sample}}_{s} = {VaccineProbability}_{{AG}_{i};R_{j};W_{k}}$$

*In-silico* competition assay description:

The *in-silico* competition assay was based on a modified version of the SIR model to test the ability of different viral variants to compete until saturation for a limited set of hosts. Since the goal of the model was to evaluate the fitness of different viruses, no recovery was implemented in the model. In the model four compartments were included: susceptible-vaccinated, susceptible-unvaccinated, infected-vaccinated, and infected-unvaccinated. The transitions between susceptible and infected were modelled using eight differential equations (*Eq. 1* to *Eq. 8*) and six ordinary equations (*Eq. 9* to *Eq. 14*). To model the expansion of the viruses, we decomposed the fitness of the virus into basal fitness, transmissibility on the vaccinated, transmissibility on the unvaccinated and vaccine escape fraction. Except for the transmissibility on the unvaccinated which was fixed to one, the other parameters (I.e., basal fitness, transmissibility on the vaccinated, and vaccine escape fraction) were randomly assigned for each simulation. See Table S1 for the ranges of the coefficients.

For each set of coefficients, there were simulated nine vaccination scenarios ranging from 10% to 90% of the population vaccinated. The nine different scenarios were then used to estimate the correlation between the expansion of V0, V1 and V2 and the vaccination. To do that, we calculated the slope of a linear regression model between the ratio of vaccinated and the ratio of each virus at the final time step. Positive slopes mean that the virus was expanding as the vaccinated population increased and negative slopes mean that the virus declined as the vaccination increased. The sum of the slopes for the three viruses for each combination of coefficients was approximately cero, meaning that as one virus declined, another one took over. Although, we noticed that the sum of slopes was not exactly cero (average sum of the slopes 1.11e-15), we considered that it was probably due to very small uncertainties during the modelling and slope calculations.

We did not consider decay of the vaccine effect over time or changes in the vaccination ratio. Therefore, we did not implement any transition between the vaccinated and unvaccinated compartments.

We manually adjusted the population size, time range and basal fitness so that the simulations had a good dynamic range, they were stable at the final time and had affordable computation time. See Table S1 for the population size and time range used in the simulations.

To speed up the computations, we parallelize the computations at the highest level. The individual processes parallelized included the nine simulations considering the different vaccination scenarios. We parallelized the models using the R packages *deSolve*, *doParallel*, *foreach*, *doSNOW* and *parallel* and we modelled the differential equations using the *deSolve* package in R. The code to run the simulations is available on the Github repository <https://github.com/garcia-nacho/3VirusSim>

Equations for the in-silico competition assay:

*Eq. 1:*

$$\frac{dSV}{dt}=-SV\cdot V1_{v}\cdot V1Tra_{v}\cdot V1Inf_{v} - SV\cdot V1_{nv}\cdot V1Tra_{nv}\cdot V1Inf_{v} -SV\cdot V2_{v}\cdot V2Tra_{v}\cdot V2Inf_{v} - SV\cdot V2_{nv}\cdot V2Tra_{nv}\cdot V2Inf_{v} -SV\cdot V3_{v}\cdot V3Tra_{v}\cdot V3Inf_{v} - SV\cdot V3_{nv}\cdot V3Tra_{nv}\cdot V3Inf_{v}$$

*Eq. 2: Susceptible non-vaccinated*

$$\frac{dSNV}{dt}=-SNV\cdot V1_{v}\cdot V1Tra_{v}\cdot V1Inf_{nv} - SNV\cdot V1_{nv}\cdot V1Tra_{nv}\cdot V1Inf_{nv} -SNV\cdot V2_{v}\cdot V2Tra_{nv}\cdot V2Inf_{nv} - SNV\cdot V2_{nv}\cdot V2Tra_{nv}\cdot V2Inf_{nv} -SNV\cdot V3_{v}\cdot V3Tra_{v}\cdot V3Inf_{nv} - SNV\cdot V3_{nv}\cdot V3Tra_{nv}\cdot V3Inf_{nv}$$

*Eq. 3:*

$$\frac{dVV1}{dt}=SV\cdot V1_{v}\cdot V1Tra_{v}\cdot V1Inf_{v} + SV\cdot V1_{nv}\cdot V1Tra_{nv}\cdot V1Inf_{v}$$

*Eq. 4:*

$$\frac{dNVV1}{dt}=SNV\cdot V1_{v}\cdot V1Tra_{v}\cdot V1Inf_{nv} + SNV\cdot V1_{nv}\cdot V1Tra_{nv}\cdot V1Inf_{nv}$$

*Eq. 5:*

$$\frac{dVV2}{dt}=SV\cdot V2_{v}\cdot V2Tra_{v}\cdot V2Inf_{v} + SV\cdot V2_{nv}\cdot V2Tra_{nv}\cdot V2Inf_{v}$$

*Eq. 6:*

$$\frac{dNVV2}{dt}=SNV\cdot V2_{v}\cdot V2Tra_{v}\cdot V2Inf_{nv} + SNV\cdot V2_{nv}\cdot V2Tra_{nv}\cdot V2Inf_{nv}$$

*Eq. 7:*

$$\frac{dVV0}{dt}=SV\cdot V0\cdot V0Tra_{v}\cdot V0Inf_{v} + SV\cdot V0_{nv}\cdot V0Tra_{nv}\cdot V0Inf_{v}$$

*Eq. 8:*

$$\frac{dNVV0}{dt}=SNV\cdot V0_{v}\cdot V0Tra_{v}\cdot V0Inf_{nv} + SNV\cdot V0_{nv}\cdot V0Tra_{nv}\cdot V0Inf_{nv}$$

*Eq. 9:*

$$V0Inf_{nv} =BaseV0$$

*Eq. 10:*

$$V0Inf_{v} =BaseV0\cdot V0Escape$$

*Eq. 11:*

$$V1Inf_{nv} =BaseV1$$

*Eq. 12:*

$$V1Inf_{v} =BaseV1\cdot V1Escape$$

*Eq. 13:*

$$V2Inf_{nv} =BaseV2$$

*Eq. 14:*

$$V2Inf_{v} =BaseV2 \cdot V2Escape$$

Where the dependent variables *SV(t),* *SNV(t)*, *VV0(t)*, *NVV0(t)*, *VV1(t)*, *NVV1(t)*, *VV2(t)*, *NVV2(t)* are respectively the number of susceptible vaccinated individuals, susceptible unvaccinated individuals, vaccinated individuals infected with the control virus V0, unvaccinated individuals infected with the control virus V0, vaccinated individuals infected with the early virus V1, unvaccinated individuals infected with the early virus V1, vaccinated individuals infected with the late virus V2 and unvaccinated individuals infected with the late virus V2 at time *t*.

*BaseV0*, *BaseV1* and *BaseV2* are the basal-fitness coefficients for the variants V0, V1 and V2 respectively.

*V0Escape*, *V1Escape* and *V2Escape* are the *vaccine-escape-fraction* coefficients for the variants V0, V1 and V2 respectively.

*V0Tra_v_*, *V0Tra_nv_*, *V1Tra_v_*, *V1Tra_nv_*, *V2Tra_v_*, *V2Tra_nv_* are the transmissibility coefficients on the vaccinated (subscript *v*) and unvaccinated (subscript *nv*) for the variants V0, V1 and V2 respectively.
