# Supplementary material for: Tracing the adaptive evolution of SARS-CoV-2 during vaccine roll-out in Norway
Source: Virus Evol. 2023 Dec 20;10(1):vead081. doi: 10.1093/ve/vead081 (PMC10776306; doi:10.1093/ve/vead081)
Supplement: vead081_Supp [file vead081_supp.zip › suppl_data/Supplementary Table 1.docx]

**Table S1.** Coefficients and parameters used during the simulations of scenarios.

|  | **V0** | **V1** | **V2** |
| --- | --- | --- | --- |
| **Basal Fitness** | 0.002-0.01 | 0.002-0.01 | 0.002-0.01 |
| **Relative Basal Fitness** | 0.2-1 | 0.2-1 | 0.2-1 |
| **Vaccine Escape Fraction** | 0-1 | 0-1 | 0-1 |
| **Transmissibility vaccinated** | 0-1 | 0-1 | 0-1 |
| **Transmissibility unvaccinated** | 1 | 1 | 1 |
| **Starting count unvaccinated** | 20 | 20 | 2 |
| **Starting count vaccinated** | 0 | 0 | 0 |
| **Population size** | 50000 | | |
| **Time steps** | 401 | | |
| **Vaccination ranges** | 0.1-0.9 | | |
